# Cyanobacteria and Soil Restoration: Bridging Molecular Insights with Practical Solutions

**DOI:** 10.3390/microorganisms13071468

**Published:** 2025-06-24

**Authors:** Matias Garcia, Pablo Bruna, Paola Duran, Michel Abanto

**Affiliations:** 1 Programa de Doctorado en Ciencias Mención Biología Celular y Molecular Aplicada, Universidad de La Frontera, Temuco 01145, Chile; mdagarcia07@gmail.com; 2 Biocontrol Research Laboratory, Universidad de La Frontera, Temuco 01145, Chile; p.bruna01@ufromail.cl; 3 Scientific and Technological Bioresource Nucleus (BIOREN), Universidad de La Frontera, Temuco 01145, Chile; 4 Departamento de Producción Agropecuaria, Facultad de Ciencias Agropecuarias y Medioambiente, Temuco 01145, Chile

**Keywords:** biocrusts, microbial ecology, metagenomics, ecology, climate change

## Abstract

Soil degradation has been accelerating globally due to climate change, which threatens food production, biodiversity, and ecosystem balance. Traditional soil restoration strategies are often expensive, slow, or unsustainable in the long term. In this context, cyanobacteria have emerged as promising biotechnological alternatives, being the only prokaryotes capable of performing oxygenic photosynthesis. Moreover, they can capture atmospheric carbon and nitrogen, release exopolysaccharides (EPSs) that stabilize the soil, and facilitate the development of biological soil crusts (biocrusts). In recent years, the convergence of multi-omics tools, such as metagenomics, metatranscriptomics, and metabolomics, has advanced our understanding of cyanobacterial dynamics, their metabolic potential, and symbiotic interactions with microbial consortia, as exemplified by the cyanosphere of *Microcoleus vaginatus.* In addition, recent advances in bioinformatics have enabled high-resolution taxonomic and functional profiling of environmental samples, facilitating the identification and prediction of resilient microorganisms suited to challenging degraded soils. These tools also allow for the prediction of biosynthetic gene clusters and the detection of prophages or cyanophages within microbiomes, offering a novel approach to enhance carbon sequestration in dry and nutrient-poor soils. This review synthesizes the latest findings and proposes a roadmap for the translation of molecular-level knowledge into scalable biotechnological strategies for soil restoration. We discuss approaches ranging from the use of native biocrust strains to the exploration of cyanophages with the potential to enhance cyanobacterial photosynthetic activity. By bridging ecological functions with cutting-edge omics technologies, this study highlights the critical role of cyanobacteria as a nature-based solution for climate-smart soil management in degraded and arid ecosystems.

## 1. Introduction

Degraded soil is defined as “the long-term reduction or loss of biological and economic productivity due to natural or anthropogenic processes, including erosion, salinization, compaction, acidification, and organic matter loss” [1]. This degradation poses a major challenge for agriculture, as it is estimated that 38% of agricultural soils are currently degraded, and by 2050, this percentage can reach 90% [2]. This phenomenon is primarily driven by climate change, increased greenhouse gas emissions, and poor agricultural practices [3]. The decline in agricultural soils poses significant challenges for global food production because 60% of the global food supply depends on soil health. For example, it has been predicted that food production will decrease by up to 30% over the next 20 years [4,5]. Furthermore, studies have reported a decline in protein content in legumes and a reduction in essential mineral nutrients in vegetables in the United States, which is attributed to soil degradation and nutrient depletion [6,7].

In this context, microorganisms have recently gained attention for their potential role in mitigating nutrient depletion in soils because they are intrinsically linked to biogeochemical cycles of essential elements such as carbon, phosphorus, and nitrogen [8,9]. Cyanobacteria are key microbial engineers owing to their unique physiological traits and ecological versatility. As the only prokaryotes capable of oxygenic photosynthesis, they contribute significantly to carbon sequestration and nitrogen fixation, thereby enhancing nutrient availability in nutrient-depleted soils [10]. Moreover, many cyanobacterial strains produce extracellular polymeric substances (EPS) that bind soil particles, improve water retention, stabilize the soil matrix, reduce erosion, and enhance the soil structure [11,12].

Cyanobacteria also exhibit high resilience to environmental stresses, such as desiccation, UV radiation, and salinity, allowing them to thrive in degraded and arid soils [13,14,15]. Their metabolic plasticity enables rapid colonization of disturbed soils, where they often initiate biological soil crust (biocrust) formation, a process that fosters microbial succession and paves the way for vegetation recovery [16,17]. Studies have shown that inoculation of degraded soils with cyanobacteria can lead to improvements in soil aggregation, fertility, and microbial diversity, ultimately contributing to ecosystem resilience and restoration [18,19].

**Figure 1:**
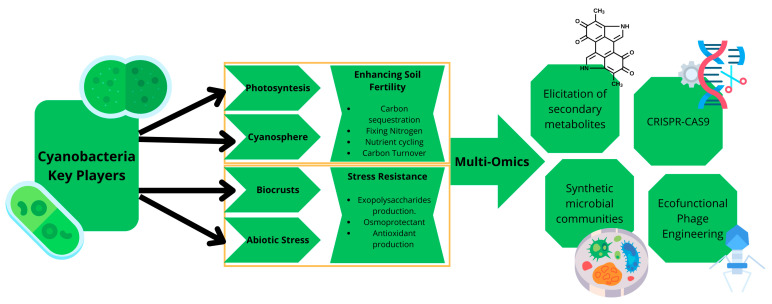
Cyanobacteria, as key players, contribute to soil fertility through photosynthesis and interactions with microbial communities (*cyanosphere*). Additionally, they form biocrusts that enhance soil structure and exhibit resilience to abiotic stresses such as salinity and desiccation, through the production of extracellular polymeric substances (EPS), compatible solutes, and antioxidant compounds. The integration of multi-omics tools enables the exploration of their functional potential, the identification of secondary metabolites, the prediction of synthetic microbial communities, the application of CRISPR-Cas9 technologies, and the design of ecofunctional phage engineering strategies.

This review aims to compile studies that have explored the use of cyanobacteria for potential applications in soil restoration and examine current relevant approaches, such as sequencing technologies applied to both isolated strains and samples containing cyanobacterial communities, including biocrusts (Figure 1). Although numerous publications have addressed the ecological role of cyanobacteria, few have integrated a multi-omics perspective with practical applications for soil restoration in the context of climate change. This review seeks to fill this gap by proposing a conceptual and technological framework for practical implementation.

## 2. Cyanobacteria: Key Players for Improving Heal Soils

Cyanobacteria are widely distributed microorganisms found in both terrestrial and aquatic ecosystems, including the Antarctic region [20]. These Gram-negative bacteria, commonly referred to as “blue-green algae” [21], were the first organisms capable of converting light energy into chemical energy through photosynthesis. They exhibit significant diversity in both unicellular and filamentous forms. As primary producers at the base of the food chain, they serve as food sources for herbivorous species, such as zooplankton [22]. Their inherent adaptability to diverse habitats, especially extreme environments, is attributed to effective stress-protection mechanisms. These characteristics explain their presence in ecosystems ranging from polar regions to arid lands, including the formation of biological soil crusts in deserts, including Antarctica and highly arid regions, such as biological soil crusts in deserts [23,24].

### 2.1. Photosynthesis, Carbon Sequestration, and Nitrogen Fixation: Enhancing Soil Fertility

Cyanobacteria are the only prokaryotes capable of performing oxygenic photosynthesis, using the Calvin–Benson–Bassham cycle to fix atmospheric carbon dioxide. Unlike other photosynthetic organisms, they possess a specialized structure called the carboxysome, which concentrates CO_2_ around the enzyme ribulose-1,5-bisphosphate carboxylase/oxygenase (RuBisCO)—a key enzyme responsible for catalyzing the first major step of carbon fixation. Although RuBisCO is widespread among autotrophs, it is inherently inefficient and prone to fixing O_2_ instead of CO_2_ [25,26]. By localizing RuBisCO within carboxysomes, cyanobacteria significantly improve its catalytic efficiency compared to terrestrial plants [27,28]. Although their light-to-biomass conversion efficiency typically ranges between 1% and 3%, under optimal conditions it can approach the theoretical limit of 12% [29,30]. Furthermore, their ability to thrive in extreme environments—including arid, nutrient-poor, and saline soils—makes them ideal candidates for ecological applications without competing with agricultural land. For instance, *Cyanobacterium aponinum* UTEX 3222 can grow under high salinity, elevated CO_2_, and low pH conditions [31]. Their metabolism contributes not only to climate change mitigation but also to reducing the rate of carbon turnover, enhancing carbon retention in the soil [32]. However, processes such as the priming effect, a phenomenon in which the input of fresh organic carbon (e.g., photosynthates) into soil stimulates microbial activity, leading to increased decomposition of older, more stable carbon pools, may be triggered by the release of photosynthates into the soil, stimulating heterotrophic microorganisms—such as nitrogen-fixing diazotrophs—to consume both recent and older carbon sources, potentially accelerating carbon loss [33,34]. Several carbon fixation pathways in cyanobacteria, especially the Calvin cycle, involve key genes directly linked to carbon capture and stabilization. Recent studies have proposed that silencing or modifying certain genes may reduce carbon turnover and increase soil carbon storage, although such strategies have yet to be validated in natural soils or pioneer species [35,36]. It has been demonstrated that there is a close relationship between these processes, where increased availability of inorganic carbon enhances the photosynthetic rate and carbohydrate synthesis. This, in turn, provides more ATP and reduces agents such as NADPH, which indirectly promotes nitrogen fixation catalyzed by the nitrogenase enzyme [37,38].

On the other hand, metabolic engineering has led to improvements in photosynthetic performance and the production of valuable compounds such as polyhydroxyalkanoates and biofuels [39,40,41]. Moreover, enhancing EPS production may represent a crucial target for improving soil structure and resilience in degraded or aggregation-deficient soils [42].

### 2.2. EPS and Biocrust Formation: Improving Soil Structure and Stability

Cyanobacteria play a crucial role in the formation and function of biological soil crusts (biocrusts). Located just a few centimeters above the soil surface, biocrusts form a rough, often dark, or charred-looking layer that spreads between shrubs and grasses in arid lands [43]. They are essentially the “skin of the desert,” a complex community of lichens, mosses, and cyanobacteria that thrive on the surface of dryland soils [44]. Remarkably, this intricate microbial assemblage plays a significant role in maintaining the entire ecosystem of soils exposed to drought and contributes to safeguarding human health [45].

These photosynthetic bacteria are the primary components of biocrusts, along with other microorganisms such as fungi, microalgae, and archaea [46]. As cyanobacteria grow and develop, they form a structural network that enhances soil cohesion, which is beneficial in erosion-prone areas. The biocrust structure plays a crucial role in preventing soil loss and degradation, contributing to the stability and sustainability of arid and semiarid ecosystems [47,48]. Another crucial aspect of cyanobacteria in biocrusts is their ability to store carbon and fix nitrogen [49,50], which has a positive impact on soil fertility and plant health [51]. Their contribution to biocrusts is further supported by their adaptations that enhance soil water retention. This, in turn, facilitates the release of EPS, which forms a gel-like matrix that retains soil moisture, making it available to plants and other organisms. Cyanobacteria provide a stable and favorable foundation for the colonization of other microorganisms, further contributing to soil stability and recovery of degraded areas [52,53]. Moreover, EPS production enhances microbial resilience, influences microbial–environment interactions, and serves as a substrate for microbial growth [54].

Soil cyanobacteria (e.g., *Nostoc*, *Microcoleus*, *Scytonema*, and others) produce EPS that perform crucial ecological functions [55]. However, their natural EPS production tends to be limited; therefore, recent research has employed multi-omics approaches and gene editing to understand and enhance EPS synthesis in these cyanobacteria.

For example, researchers have evaluated various culture conditions for *Synechocystis* sp. PCC 6803, which affect both the quantity and composition of EPS, along with the transcriptomic profile. The modification of mineral nutrition significantly alters EPS production. For instance, reducing Mg increased EPS productivity per cell, and there was a correlation between sulfate levels and higher xylose content in the produced EPSs [56]. This study shows that EPS production and types of EPS can be adjusted through environmental conditions and metabolic engineering strategies to redirect cellular resources for EPS synthesis. In this case, the composition of EPSs is xylose, but in others, it could change the composition with another monosaccharide, such as galactose, mannose, and rhamnose [57]. A similar situation applies to *Nostoc flagelliforme*, where researchers exposed the organism to different light conditions and used an iTRAQ-based proteomic analysis to precisely identify and quantify proteins, along with functional classification to determine which processes these proteins are involved in. They concluded that different light types can stimulate *N. flagelliforme* to produce polysaccharides by modifying carbon metabolism and diverting more carbon toward the synthesis of sugar precursors [58]. Similarly, environmental conditions can be manipulated to increase the production of biotechnologically relevant compounds, as supported by multi-omics approaches [59]. In addition to manipulating environmental conditions, researchers have explored environmental elicitation strategies to induce EPS overproduction, which is particularly relevant for applications in deserts or degraded soils. For example, exposing *N. flagelliforme* to 12 different compounds revealed that salicylic acid and jasmonic acid significantly increased EPS synthesis by approximately 20% compared to the control [60]. Importantly, these chemical elicitors raised EPS levels without altering their structure, unlike certain stress factors such as UV radiation, drought, pH, or extreme temperatures, which can change EPS composition [61].

The integration of multi-omics tools has clarified the genes and conditions involved in polysaccharide synthesis, leading to substantial advances. Combining these approaches with gene-editing techniques such as CRISPR-Cas could allow us to promote biocrust formation in desert, degraded, or burned soils for restoration purposes [62,63]. However, more studies are needed because EPS overproduction and biocrust formation vary depending on the cyanobacterial strain and soil type. Moreover, while editing a prokaryote is much more feasible than editing a higher plant or eukaryotic microalga, genetic editing in cyanobacteria is not as advanced as in a model microorganism such as *E. coli*.

### 2.3. Stress Resilience and Functional Adaptability in Harsh Environments

These pioneer microorganisms can survive for prolonged periods without water, high salinity levels, and intense solar radiation owing to their unique molecular adaptations. In recent years, researchers have integrated multiple disciplines, such as multi-omics approaches, genetic engineering, and microbial consortia, to enhance the stress tolerance of cyanobacteria, with promising applications in soil restoration and resilient agriculture.

#### 2.3.1. Desiccation Tolerance and Protective Genes

Anhydrobiotic cyanobacteria possess the remarkable ability to survive with less than 10% cellular water, due to a complex orchestration of molecular mechanisms [64]. A transcriptomic study on *Chroococcidiopsis* sp. (desert strain ABS-02) revealed the activation of antioxidant genes that counteract oxidative stress during drought conditions [65]. This strain exhibited a rapid recovery of photosystem II activity following rehydration, associated with the synthesis of EPSs and sucrose, which help reduce water loss and stabilize cellular osmolarity [66]. Moreover, it showed increased accumulation of trehalose, a key compatible solute, which is a non-reducing disaccharide formed by two glucose units that plays a crucial role in stabilizing cellular structures and preserving enzyme function under osmotic and desiccation stress [67], while simultaneously suppressing metabolic activity and activating energy-saving pathways to withstand the dry state. Resilient strains such as *Chroococcidiopsis* sp. are of vital importance in the study of life in extreme environments. The quantification of osmoprotectants through metabolomic approaches, combined with transcriptomic analysis of genes encoding antioxidant enzymes, provides insights into their desiccation response. Nevertheless, although axenic studies are essential, it is equally important to assess their behavior in microbial consortia or co-cultures. This would help determine whether their adaptive capacities can be shared with surrounding microbiomes, fostering community-level resilience under environmental stress.

Cyanobacteria exhibit remarkable adaptations during the quiescent phase, enabling them to endure extreme environmental conditions. This state, known as dormancy, is a reversible metabolic shutdown that allows cells to survive unfavorable conditions by minimizing energy expenditure and halting growth [68]. During these periods, cyanobacteria develop resilience to stressors, such as UV radiation, by producing photoprotective compounds, like scytonemin, as well as antioxidants that facilitate DNA repair [21,69]. However, these metabolites are synthesized only when cells become hydrated and initiate metabolic activity. Consequently, although these protective compounds remain present during dormancy, cyanobacteria cease their active production of trehalose. Additionally, they suspend photosynthesis to reduce oxidative stress and prepare for desiccation, a process predicted by phytochromes that sense UV radiation and activate all these mechanisms [70,71]. Moreover, EPSs play a crucial role not only in soil aggregation but also in reducing water loss among microorganisms within biocrusts. This function extends the duration of metabolite release and enhances cyanobacteria’s ability to withstand prolonged dormancy [71]. Additionally, previous studies have demonstrated that a reduction in EPS production is directly linked to decreased moisture retention. For instance, in *Nostoc flagelliforme*, a gene cluster comprising four genes regulated by an *RpaB-like* transcription factor was identified as essential for desiccation tolerance. When this cluster was knocked out, the strain exhibited a significant loss in its ability to tolerate desiccation. Conversely, heterologous expression of this gene cluster in *Nostoc* sp. PCC 7120 enhanced its desiccation resistance [72], highlighting the potential of transferring co-evolved genes from novel cyanobacterial strains into a well-studied model through genetic engineering.

Drought-related studies have been extensively conducted in the context of plant–soil interactions, where it has been observed that plants under drought stress can trigger a mechanism known as “cry for help” [73]. Through this response, plants release specific chemical signals in the rhizosphere to recruit beneficial microorganisms that enhance their drought tolerance. Could cyanobacteria exhibit a similar strategy? It is plausible that soil-dwelling cyanobacteria might release exopolysaccharides or other organic compounds capable of attracting microbial partners to help them withstand drought conditions. However, this potential mechanism remains poorly understood and warrants further investigation using both metagenomic and metabolomic approaches specifically tailored to drought stress scenarios.

#### 2.3.2. Salinity Resistance

Salinity stress represents a common challenge in degraded soils because salt accumulation disrupts soil structure, reduces water availability, and limits microbial and plant activity [74,75,76]; however, the production of compatible solutes such as sucrose, glycerol, glucosides, ectoine, and proline can counteract osmotic pressure [77,78]. Among these, ectoine has been extensively studied: for instance, a mutant strain of *Synechococcus elongatus* PCC 7942 expressing ectoine biosynthetic genes was able to survive at 500 mM NaCl, a condition where the wild-type strain could not persist [79]. Another approach involves the exogenous supplementation of solutes; in *Arthrospira platensis*, trehalose addition under nutrient stress increased glycogen content by 54%, thereby alleviating stress and activating cellular preservation pathways. This strategy has prompted further research aiming to enhance endogenous osmoprotectant production through genetic engineering, with CRISPR-based applications achieving up to 80% increases in vitro [80]. Microbial consortia have also shown promising results; co-cultivation of the potassium-solubilizing bacterium *Paenibacillus sabinae* with the cyanobacterium *Leptolyngbya* sp. RBD05 led to an 85% increase in wheat dry biomass, attributed to a synergistic halotolerant association that enhanced nitrogen availability (via *Leptolyngbya* sp.) and potassium uptake (via *P. sabinae*) [81]. Hardening, on the other hand, represents a complementary approach to those previously described. For instance, pre-cultivation under moderate osmotic stress improved subsequent tolerance to desiccation and UV stress. This treatment induced *Stenomitos frigidus* to increase the production of EPSs, carotenoids, and photoprotective compounds (sunscreens), as well as to accumulate trehalose and sucrose under dry soil conditions [59]. These findings highlight how diverse strategies can converge to build resilience in saline-affected soils; furthermore, while *Leptolyngbya* is a non-heterocystous cyanobacterium, future studies could explore whether heterocystous nitrogen-fixing strains may engage in direct symbiosis with *P. sabinae*, potentially boosting antioxidant activity or proline production in this halophilic bacterium.

### 2.4. Microbial Interactions in the Cyanosphere: Promoting Ecosystem Function

Cyanobacteria, like all microorganisms, are never solitary; they are always in constant symbiosis with other microorganisms, fungi, and even plants. In the special case of cyanobacteria, they create a zone in which they release photosynthates and nutrients specific to the development of microbial life, called the cyanosphere [82], which is analogous to the term rhizosphere in plants. In a soil restoration scenario, the cyanosphere environment is a key point because it requires a certain richness and abundance of microorganisms present [83], which are responsible for fulfilling different functions to make the soil suitable for cultivation. Cyanobacteria, thanks to their release of EPSs [84], make this microenvironment habitable for various diazotrophic microorganisms [85]. However, the inoculation of cyanobacteria has faced several challenges in achieving successful colonization in dry soils [86].

**Figure 2:**
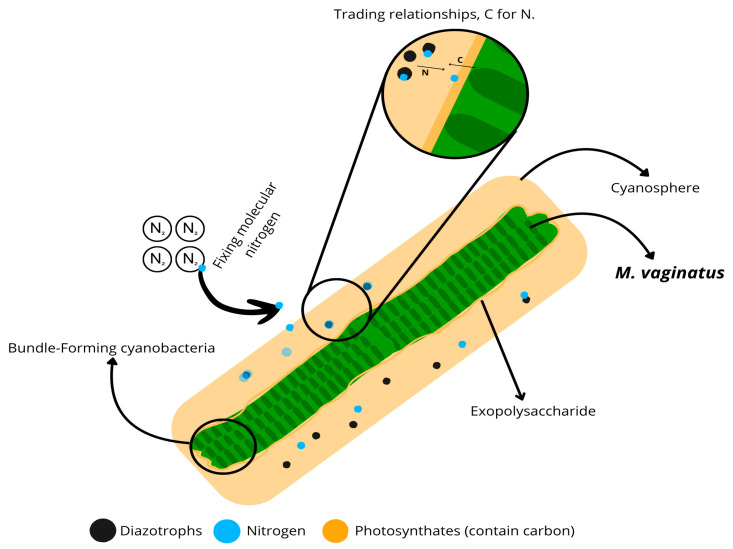
Nitrogen fixation and carbon exchange in the cyanosphere. This schematic representation illustrates the interactions within the cyanosphere, highlighting the role of bundle-forming cyanobacteria, such as *Microcoleus vaginatus*, in nitrogen fixation and carbon exchange. Diazotrophic bacteria (black dots) fix atmospheric nitrogen (N_2_) and release bioavailable nitrogen (blue dots), while photosynthates of cyanobacteria (orange) provide carbon-rich compounds through EPSs. The inset magnifies microbial trading relationships, showing the exchange of carbon (C) for nitrogen (N) within this microbial community.

The release of EPSs is well documented, with various enzymes involved in the synthesis of these sugars [87]. By analyzing enzymatic profiles alongside cyanobacterial genomics, it would be possible to identify potential strains capable of producing sufficient EPSs to trigger a priming effect in nutrient-deficient soils, while simultaneously promoting the establishment of a known biocrust species. For instance, *M. vaginatus* is a filamentous cyanobacterium whose filaments align in parallel and form continuous bundles. This structural arrangement optimizes resource utilization, particularly during the release of EPSs and photosynthates, thereby increasing available carbon within the cyanosphere. Consequently, heterotrophic diazotrophic microorganisms can utilize this carbon to enhance their fitness and fix nitrogen. This mutualistic function between *M. vaginatus* and microorganisms within its cyanosphere enables the survival of soils with low nutrient levels or degraded conditions, ultimately fostering the formation of a viable biocrust capable of supporting plant cultivation (Figure 2).

Currently, field microbiology relies on multi-omics approaches to characterize and find answers regarding the stabilization of cyanobacteria in soils [88,89]. However, this requires a multidisciplinary field that enables a precise understanding of the soil to be treated. Moreover, applying meta-omics approaches (metagenomics, metatranscriptomics, metaproteomics) in environments like soil is often challenging, as these environments depend on numerous external parameters such as soil type, chemical parameters (pH, Total Carbon, Total Nitrogen, among others), moisture, and climate. In the specific case of metatranscriptomics, the “snapshot” taken from a given soil may no longer be accurate after several months [90,91]. Researchers should first seek pragmatic solutions that go beyond direct inoculation, such as using vegetative mesh cover or microbial consortia that enable better treatment [92,93]. From there, multi-omics approaches can be applied to characterize the solution, as performing the reverse would lead to a waste of resources.

Thanks to meta-omics technologies, researchers have been able to specifically investigate the microbiome surrounding cyanobacteria [94,95]. Evidence suggests the presence of a core microbiome within cyanobacterial strains, which may vary depending on the environmental sources. Cyanobacterial species might recruit specific microorganisms for their benefit, helping them withstand stressful environments. However, this phenomenon has been extensively studied in aquatic ecosystems. Therefore, it would be highly impactful to “train” cyanobacteria to recruit microorganisms into their cyanosphere that can thrive in dry or nutrient-depleted soils before inoculation. This should be evaluated using omics technologies both before and after inoculation [96,97,98]. Additionally, special attention has been given to the mobile genetic elements of cyanobacteria. It has been shown that a significant part of the functional role of cyanobacterial communities is mediated by horizontal gene transfer, whether through plasmids, insertion sequences, or prophages [98]. While cyanophages have been studied in marine samples, emphasis should also be placed on the prophages found in challenging soil environments, such as drylands, biocrusts, or soils undergoing pre- and post-restoration. Although microbial communities shift in composition, microbiomes have a profound impact that appears to be immobilized in microbiome regulation.

## 3. The Role of Sequencing Technologies in Understanding Cyanobacteria Dynamics in Soils

The rapid advancement of sequencing technologies has enabled the study of how cyanobacteria interact within the soil microenvironment at the community level with the aim of facilitating soil restoration. Metagenomics approaches provide insights into the interactions between various microorganisms in extreme environments, allowing researchers to understand how these microbiomes may alter their diversity in response to climate change [99]. However, much of the functional role of cyanobacteria in microenvironments under abiotic stresses, such as desiccation and high temperatures, remains an enigma. For instance, nitrogen fixation genes can be detected in biocrusts, yet young biocrusts are typically dominated by non-heterocystous cyanobacteria [100,101]. This suggests that the activity of these genes is not performed by cyanobacteria but rather by other diazotrophic bacteria. This highlights the importance of studying not only cyanobacteria-based taxonomy and functionality but also the surrounding microbiome consortium [102]. To address these knowledge gaps, deeper metagenomic shotgun sequencing should be conducted to identify specific genes present in each cyanosphere. Additionally, metabarcoding-based studies should not be disregarded, as they provide essential information on microbiome taxonomy and modulation under conditions such as hydration, organic matter aggregation, and increased CO_2_ levels in soil samples [103]. These studies would complement metagenomic analyses, leading to a more comprehensive understanding of the microbial interactions in these environments.

Focusing on the isolation of cyanobacteria reveals numerous potential applications that can be explored using both genomics and metabolomics. However, a major limitation of genomic studies is that cyanobacteria typically function within consortia and engage in mutualistic relationships with other bacteria [104]. As a result, obtaining an axenic culture of a single strain is nearly impossible in many cases. Moreover, it has been demonstrated that in the absence of essential coexisting bacteria, cyanobacteria not only exhibit insufficient growth but also fail to produce the metabolites necessary for survival [82]. Thus, sequencing cyanobacteria within a consortium-based framework may be necessary, utilizing a hybrid assembly approach that considers both the cyanobacterium and bacterial community present in its cyanosphere. Investigating cyanobacteria across various soil types can provide valuable insights into their inoculation, and with the aid of advanced sequencing technologies, crucial knowledge can be obtained for applications in soil restoration (Table 1) [105,106].

### Unlocking the Biotechnological Potential of Cyanobacteria with Bioinformatics Tools

Current bioinformatics tools have represented a breakthrough for modern biotechnology, as they have enabled a deeper exploration of the functional potential of various organisms, including environmental microorganisms [137]. Through these tools, multiple applications have been identified across diverse areas of biotechnology. In fact, access to open-source and freely available databases allows researchers to work with cyanobacterial genomes from studies conducted worldwide, like a CyanoCyc cyanobacterial web portal that integrates a rich database collection about cyanobacterial genomes with an extensive suite of bioinformatics tools (https://cyanocyc.org) [138]. It is possible to infer the metabolic pathways present in the studied cyanobacteria using tools such as KEGG, KO, and other similar platforms [139], which are freely accessible and user-friendly, thus making them available to any researcher [140]. In addition to tools that assess metabolic functionality, the production of secondary metabolites in cyanobacteria can also be investigated through the analysis of biosynthetic gene clusters. Moreover, there are open-access databases specifically dedicated to secondary metabolites produced by cyanobacteria, which facilitate their identification and characterization [141]. These genetic groupings allow for the determination of the biosynthetic capacity for compounds such as compatible solutes, including ectoine and proline, which are capable of conferring resilience in arid soils. To carry out this analysis, the freely accessible tool AntiSMASH v8.0 can be used [142], which is available online and allows the prediction of such clusters from the input genome. For comparative and exploratory studies, especially those involving horizontal gene transfer, multiple platforms are available to identify CRISPR arrays, such as CRISPRDetect v3.0 [143]. Additionally, for the identification of environmental prophages, the most precise (fewer false positives) tools [144] include VirSorter2 [145] and VIBRANT v1.2.1 [146].

In recent years, artificial intelligence has gained significant relevance, revolutionizing science and expanding its applications across various fields. One of the most well-known tools is AlphaFold v3.0, developed by DeepMind [147], which successfully predicted with high accuracy the three-dimensional structure of proteins from their amino acid sequences, thus solving a problem that had remained unresolved for decades. Thanks to this tool, it is no longer necessary to wait years to obtain the structure of a protein; instead, it can now be achieved in a matter of minutes. This application is entirely novel in the fields of bioengineering and metabolic optimization, particularly for environmental microorganisms such as cyanobacteria, which are highly diverse and understudied in extreme environments [148]. As novel organisms, many of their proteins are poorly annotated or classified as hypothetical [149,150]. In this context, AlphaFold would allow us to discover new enzymes resistant to osmotic stress, carbon and nitrogen transport proteins, and/or exopolysaccharide regulators that could enhance soil aggregation. Moreover, it has been demonstrated that AlphaFold is not only useful for structure prediction but that its combination with other tools, such as Foldseek clustering a platform that groups proteins with similar three-dimensional structures at scale—enables the analysis of protein evolution on a global scale and the inference of previously unknown functions [151].

## 4. Future Prospects and Challenges

### 4.1. State of the Art of Cyanophages’ Ability in Response to Stress and Metabolic Innovation

Cyanophages are among the viruses with the most important effects on the ecosystems [152]. These viruses infect cyanobacteria, and after infection, lytic cyanophages eventually lyse the host cell [153,154], contributing to the release of carbon and nitrites into the environment [155]. Cyanobacteria are able to fix molecular nitrogen and, as essential primary producers that channel organic carbon, play a key role in structuring the highest trophic levels in aquatic and terrestrial ecosystems. The *phoH* and *mazG* genes are widely used markers for studying marine cyanophages [156]; however, relying on them alone may underestimate the true abundance and diversity of these viruses in soil environments. As a result, our understanding of the functional roles and importance of soil-dwelling cyanobacteria is skewed [157,158]. Cyanobacteria from current systems have been shown to form blooms during warm periods of high nutrient loading, and many of these cyanobacteria decompose, sink to the eutrophic zone, and are deposited on the seafloor [154]. Cyanophages enhance the decomposition of cyanobacteria via viral lysis; however, these mechanisms have not been studied in soils. Thus, broader viromics approaches are essential for capturing the full spectrum of phage–cyanobacteria interactions, which are likely key contributors to soil ecosystem processes and resilience [157]. Applying nutrient-rich cyanobacteria—carrying carbon, nitrogen, and phosphorus—along with lysogenic phage therapy, could enhance soil restoration by providing essential nutrients for resilient microorganisms in dry and arid environments.

Auxiliary metabolic genes (AMGs) have recently attracted considerable attention because of their role in adaptive evolution. These genes, which are often acquired via horizontal gene transfer, can confer selective advantages by enabling bacteria to tolerate diverse environmental stresses. Moreover, if these genes are stably integrated into the bacterial genome, they can be transmitted through vertical gene transfer. A notable example involves marine cyanobacterial viruses (cyanophages), which have been found to carry AMGs associated with key functions, such as photosynthesis, central carbon metabolism, phosphate acquisition, and nucleotide synthesis [159]. These high diversities and abundances of AMGs suggest that phages play important roles in biogeochemical cycles. However, how much they contribute to matter and energy production remains challenging to quantify; therefore, a metagenomic mining search should be conducted to identify potential AMGs that activate metabolic genes such as photosynthesis, carbon metabolism, and nitrate reduction [160]. In paddy soils, phages have emerged as key players in carbon sequestration. Under heavy metal stress, lysogenic phage infections have been shown to upregulate auxiliary metabolic genes (AMGs) associated with carbon fixation. This positive regulation enhances the host’s carbon assimilation capacity, resulting in a 35.4% increase in microbial biomass carbon content compared to control conditions. These findings emphasize the potential role of phage–host interactions as modulators of microbial metabolic pathways, particularly in stressed environments, and highlight the utility of lysogenic phage infection in carbon-fixing microbial hosts as a strategy to enhance ecosystem-level carbon retention [161,162].

Cyanophages can operate independently by expressing photosynthesis-related genes in sunlit marine environments, thus directly influencing photophysiology beyond simply parasitizing cyanobacteria [163]. By facilitating carbon fixation, these viruses may play a pivotal role in mitigating rising greenhouse gas concentrations. Therefore, understanding cyanophage contributions to soil productivity and carbon sequestration is critical for assessing the broader ecological and climatic impacts of these viruses.

### 4.2. New Advances and Challenges in Soil Cyanobacteria Multi-Omics: Towards a Comprehensive Understanding of Their Role in Soil Restoration

Soil cyanobacteria and biocrusts have gained increasing relevance in genomics. However, significant gaps remain in these studies, particularly concerning the accuracy and quality of the available genomes (Table 1) [164,165]. Most genomes deposited in public databases originate from metagenome-assembled genomes (MAGs), which, while representing a significant improvement over the sequencing of isolated genomes, may introduce uncertainties in the identification of biosynthetic gene clusters and accurate annotation of metabolic pathways [166].

Among the most studied cyanobacteria in biocrusts are genera such as *Nostoc* and *Microcoleus*, whose surrounding microbiomes have been analyzed at the metagenomic level. However, these studies reached a bottleneck. Although microbial communities and their associated genes have been identified through rehydration and desiccation assays, the precise genes expressed during these processes remain largely unknown [167]. Consequently, an increase in multi-omics studies integrating genomics, metatranscriptomics, and/or metabolomics is expected, particularly with a focus on soil restoration using terrestrial cyanobacteria.

Due to their cellular structure and tendency to fragment during sequencing protocols based on Illumina technology, cyanobacterial MAGs often exhibit lower abundance and completeness [168,169]. Therefore, long-read sequencing technologies such as Oxford Nanopore Technologies can complement assemblies and enhance the recovery of more complete genomes [170]. From a genomic perspective, an alternative approach would be to study isolated strains in co-culture with microorganisms present in their cyanosphere, sequencing the entire consortium in a continuous manner. Additionally, in metagenomic studies, this approach allows for a more precise analysis of microbial responses within the natural environment.

## 5. Conclusions

Advancements in sequencing technologies, together with the growing availability of bioinformatics tools, have enabled the rapid characterization of various omics disciplines, allowing for targeted and context-specific analyses tailored to diverse environmental conditions. These innovations have significantly accelerated the development of solutions for soil restoration. Recent breakthroughs have unveiled key metabolic pathways in cyanobacteria, including EPS production, accumulation of compatible solutes, carbon sequestration, and nitrogen fixation. Moreover, disruptive tools such as CRISPR-Cas systems and artificial intelligence are opening new avenues for optimizing metabolic functions in cyanobacteria, thereby expanding their biotechnological applications. Investigating the integration of microbial consortia, such as the cyanosphere, represents a promising strategy, not only due to cyanobacteria–bacteria interactions but also because of horizontal gene transfer events. For example, designing an artificial consortium where heterotrophic diazotrophic microorganisms consume carbon and oxygen provided by cyanobacteria could, in return, supply them with nitrogen and CO_2_. These mobile genetic elements—including plasmids, insertion sequences, and prophages—play a crucial role in regulating microbiome functionality and enhancing its ecological and metabolic stability under extreme environmental conditions. One promising avenue is the concept of ecofunctional phage engineering, where high-nutrient cyanobacteria from eutrophic lakes could be strategically infected with lytic phages. These engineered strains may then be applied to degraded soils to improve nutrient availability and promote microbial resilience. This approach offers a novel biotechnological strategy to harness phage–host interactions for soil restoration under climate-stressed conditions. Altogether, the integrated approach—from molecular insights to field-scale applications positions cyanobacteria as key players in sustainable strategies for ecological restoration, carbon sequestration, and desertification mitigation. However, it is essential to promote further interdisciplinary studies to validate their long-term efficacy under real-world field conditions and across diverse agroecological settings.

## Figures and Tables

**Table 1:** Cyanobacterial genera for biocrust-based soil restoration *.

Genera	Application	Environmental Stress Tolerance	Soil Type	References
*Chroakolemma*	Restoration of biocrusts	UV radiation; drought; arid conditions	Phaeozem calcareous and Phaeozem (mollisol)	[107,108]
*Microcoleus*	Restoration of biocrusts and soils, and use as PGPR	Drought; high salinity; UV radiation; nutrient-poor soils.	Arid/semi-arid soils	[82,109,110,111,112,113,114,115,116,117,118,119]
*Nostoc*	Restoration of soils and biocrusts, and use as bioremediation and PGPR	Extreme temperatures; UV radiation; drought.	Arid/semi-arid soils; agricultural soils	[16,120,121,122,123,124,125,126,127,128]
*Phormidium*	Use as PGPR	High salinity; temperature fluctuations.	Arid soils; saline soils.	[129,130]
*Scytonema*	Restoration of soils and burned soils	UV radiation; drought; high temperatures; nutrient-poor soils.	Arid/semi-arid soils; burned soils	[16,50,110,114,117,118,121,122,125,126,131,132,133,134,135]
*Trichocoleus*	Restoration of soils	Drought; nutrient-poor soils.	Restoration soils (gypsiferous)	[50,126,133,136]
*Leptolyngbya*	Restoration of biocrusts	Drought; High salinity	Arid/semirad soils; Planosol	[81,135]
*Tolypothrix*	PGPR	Drought	Arid/semi-arid soils	[136]

* Overview of the main cyanobacterial genera reported in the literature, highlighting their applications (e.g., soil/biocrusts restoration, PGPR, bioremediation), tolerance to environmental stresses (UV radiation, drought, salinity, extreme temperatures, and nutrient-poor conditions), the types of soils they inhabit (arid, semi-arid, mollisol, agricultural, burned, etc.), and the corresponding references. Biocrusts = biological soil crusts; PGPR = Plant Growth Promoting Rhizobacteria.

## Data Availability

Data sharing is not applicable. No new data were created or analyzed in this study.
